# Supplementary material for: Diet and density effects on growth, proximate composition and fatty-acid profile of Hediste diversicolor juveniles
Source: PLoS One. 2026 Apr 6;21(4):e0344444. doi: 10.1371/journal.pone.0344444 (PMC13052871; doi:10.1371/journal.pone.0344444)
Supplement: Attachment — Submitted filename: PlosOne-D-25-64293-review-MN.docx [file pone.0344444.s002.docx]

The authors analysed the influence of feed type (beluga waste or commercial feed) and stocking density on the nutritional composition and growth performance of *H. diversicolor*. The experimental design is well structured, and the conclusions are supported by the results obtained. However, both the presentation of results and the statistical analysis require improvement.

**Figure 1** – In the figure, it is not clear that each experimental group corresponds to three tanks. This information should be clearly represented. Additionally, the terminology corresponding to the tested densities (i.e. 1, 2, 3 and 4) should be included.

**Line 179** – What was the feed-to-worm biomass ratio used in this study?

**Proximate composition analysis:** How was ash content determined?

**Lines 222 and 228** – Total lipids were determined using two different methods (Soxhlet and Folch). Please justify the use of two methodologies and clarify which method corresponds to the results presented.

**Line 238** – Please specify the FAME standards used.

**Line 240** – Please confirm whether fatty acid results are expressed as mg FA per g of wet tissue and explain the calculation. Tables 6, 7 and 8 present fatty acid data as a percentage of total fatty acids. How do you justify this discrepancy in units?

**Line 268** – The identification of the experimental group with a density of 5000 ind./m² (T1–3) must be included.

**Lines 298–310 and Tables 3 and 4** –
The sum of moisture, protein, lipids and ash is close to 100%. Does *H. diversicolor* not contain carbohydrates? Are these results consistent with previous studies?
Additionally, the proximate composition of *H. diversicolor* obtained with the two different feeds is not compared. The information presented in Tables 3 and 4 should be compiled into a single table. For each tested density, results obtained with the two diets should be directly compared, including appropriate statistical analysis.

**Lines 311–324 / Table 5 and Figure 3** –
Information regarding the proximate composition of the two experimental feeds is duplicated. Table 5 and Figure 3 present the same data; therefore, Figure 3 should be removed.
Moreover, the sum of moisture, protein, fat and ash represents only 53.51% and 72.21% of beluga sturgeon by-products and beluga sturgeon feed, respectively. What does the remaining fraction correspond to? Carbohydrates, including fibre? Could differences in these nutrients also help explain differences observed in polychaete performance?

**Lines 338–367** –
The fatty acid profiles of *H. diversicolor* obtained with the two feeds should be compared for each density, including statistical analysis.

**Lines 401–405** –
The data supporting these conclusions are not adequately highlighted in the Results section. Please refer to the comments regarding Tables 3 and 4.
