# Supplementary material for: Diet and density effects on growth, proximate composition and fatty-acid profile of Hediste diversicolor juveniles
Source: PLoS One. 2026 Apr 6;21(4):e0344444. doi: 10.1371/journal.pone.0344444 (PMC13052871; doi:10.1371/journal.pone.0344444)
Supplement: Attachment — Submitted filename: Response to Reviewers.docx [file pone.0344444.s004.docx]

| **Date:** | Jan 16 2026 10:08PM |
| --- | --- |
| **To:** | "Zabihollah Pajand" zpajand@gmail.com |
| **From:** | "PLOS ONE" plosone@plos.org |
| **Subject:** | PLOS ONE Decision: Revision required [PONE-D-25-64293] |
| **Attachment(s):** | [PlosOne-D-25-64293-review-MN.docx](https://www.editorialmanager.com/pone/download.aspx?guid=%7b6d5f681b-0457-4bfa-9b1e-cc9849ba49ce%7d&scheme=13&id=601689) |

PONE-D-25-64293

Diet and density effects on growth, proximate composition and fatty-acid profile of Hediste diversicolor juveniles

PLOS One

Dear Dr. Pajand,

Thank you for submitting your manuscript to PLOS ONE. After careful consideration, we feel that it has merit but does not fully meet PLOS ONE’s publication criteria as it currently stands. Therefore, we invite you to submit a revised version of the manuscript that addresses the points raised during the review process.

Please submit your revised manuscript by Mar 02 2026 11:59PM. If you will need more time than this to complete your revisions, please reply to this message or contact the journal office at [plosone@plos.org](mailto:plosone@plos.org). When you're ready to submit your revision, log on to https://www.editorialmanager.com/pone/ and select the 'Submissions Needing Revision' folder to locate your manuscript file.

Please include the following items when submitting your revised manuscript:

- A letter that responds to each point raised by the academic editor and reviewer(s). You should upload this letter as a separate file labeled 'Response to Reviewers'.
- A marked-up copy of your manuscript that highlights changes made to the original version. You should upload this as a separate file labeled 'Revised Manuscript with Track Changes'.
- An unmarked version of your revised paper without tracked changes. You should upload this as a separate file labeled 'Manuscript'.

If you would like to make changes to your financial disclosure, please include your updated statement in your cover letter. Guidelines for resubmitting your figure files are available below the reviewer comments at the end of this letter.

If applicable, we recommend that you deposit your laboratory protocols in protocols.io to enhance the reproducibility of your results. Protocols.io assigns your protocol its own identifier (DOI) so that it can be cited independently in the future. For instructions see: <https://journals.plos.org/plosone/s/submission-guidelines#loc-laboratory-protocols>. Additionally, PLOS ONE offers an option for publishing peer-reviewed Lab Protocol articles, which describe protocols hosted on protocols.io. Read more information on sharing protocols at <https://plos.org/protocols?utm_medium=editorial-email&utm_source=authorletters&utm_campaign=protocols>.

We look forward to receiving your revised manuscript.

Kind regards,

Eman Zahran, Ph.D
Academic Editor
PLOS One

Journal Requirements:\

When submitting your revision, we need you to address these additional requirements.

1.Please ensure that your manuscript meets PLOS ONE's style requirements, including those for file naming. The PLOS ONE style templates can be found at

https://journals.plos.org/plosone/s/file?id=wjVg/PLOSOne_formatting_sample_main_body.pdf and

<https://journals.plos.org/plosone/s/file?id=ba62/PLOSOne_formatting_sample_title_authors_affiliations.pdf>

2. We note that the grant information you provided in the ‘Funding Information’ and ‘Financial Disclosure’ sections do not match.

When you resubmit, please ensure that you provide the correct grant numbers for the awards you received for your study in the ‘Funding Information’ section.

3. Thank you for stating the following financial disclosure:

“This study was financially supported by grants from the Iran National Science Foundation (INSF Contract no. 91002674) program project.”

Please state what role the funders took in the study.  If the funders had no role, please state: "The funders had no role in study design, data collection and analysis, decision to publish, or preparation of the manuscript."

If this statement is not correct you must amend it as needed.

Please include this amended Role of Funder statement in your cover letter; we will change the online submission form on your behalf.

Answer: Dear Dr Zahran and the PLOS ONE Editorial Team,

Thank you for considering our manuscript titled “[**Diet and density effects on growth, proximate composition and fatty-acid profile of *Hediste diversicolor* juveniles**]” for publication in PLOS ONE, and for providing the opportunity to submit a revised version.

We sincerely appreciate the constructive feedback from the academic editor and the reviewers. We have carefully addressed all comments and requirements, as detailed below.

**1. Response to Journal Requirements:**

We have thoroughly reviewed and ensured our manuscript complies with all PLOS ONE style requirements. The revised files have been prepared and named according to the specified guidelines.

**2. Correction of Grant Information Discrepancy:**

We apologize for the oversight regarding the grant information. The correct and matching details are as follows:

- **Funding Information:** This study was supported by the Iran National Science Foundation (INSF) under grant number **91002674**.
- **Financial Disclosure:** The authors received funding from the Iran National Science Foundation (INSF Grant no. **91002674**) for this work.

These matching details have been updated in the relevant sections of the manuscript and the submission system.

**3. Role of Funder Statement:**

As confirmed in our previous correspondence, we hereby provide the amended statement as requested:

**"The funders had no role in the study design, data collection and analysis, decision to publish, or preparation of the manuscript."**

We confirm this statement is correct and authorize the journal office to update the online submission form on our behalf.

**4. Submission of Revised Materials:**

In accordance with your instructions, we have prepared and are submitting the following files:

- **Response to Reviewers:** This separate document provides a point-by-point response to all academic editor and reviewer comments.
- **Revised Manuscript with Track Changes:** A copy of the manuscript with all modifications highlighted.
- **Manuscript:** A clean version of the revised manuscript without tracked changes.

We believe the revisions have significantly strengthened the manuscript and we hope it now fully meets PLOS ONE's publication criteria.

Thank you again for your time and consideration.

Sincerely,

Pajand (On behalf of all co-authors)

4. Please note that funding information should not appear in any section or other areas of your manuscript. We will only publish funding information present in the Funding Statement section of the online submission form. Please remove any funding-related text from the manuscript.

Answer: Thanks, based on your suggestion, we removed funding information from text.

5. In the online submission form, you indicated that “The datasets used and/or analysed during the current study are available from the corresponding author on reasonable request.”

All PLOS journals now require all data underlying the findings described in their manuscript to be freely available to other researchers, either 1. In a public repository, 2. Within the manuscript itself, or 3. Uploaded as supplementary information.

This policy applies to all data except where public deposition would breach compliance with the protocol approved by your research ethics board. If your data cannot be made publicly available for ethical or legal reasons (e.g., public availability would compromise patient privacy), please explain your reasons on resubmission and your exemption request will be escalated for approval.

Answer: Thank you for your guidance regarding the PLOS ONE data availability policy.

We have now complied with your requirements as follows:

**Action Taken:** We have uploaded the complete dataset as an Excel file in the supplementary information section of our resubmission. The file is named: **"Supplementary_material.xlsx"**

**Manuscript Revision:** We have revised the **Data Availability Statement** in the manuscript accordingly. The updated statement now reads:

**"**All data generated or analysed during this study are included in this article [and its supplementary information files]. Specifically, the complete dataset is provided in S1 Dataset (Supplementary_material.xlsx).**"**

This ensures that all underlying data are freely available to other researchers in line with PLOS ONE's policy.

We hope these revisions fully address this requirement.

Sincerely,

6. If the reviewer comments include a recommendation to cite specific previously published works, please review and evaluate these publications to determine whether they are relevant and should be cited. There is no requirement to cite these works unless the editor has indicated otherwise.

Answer: Thank you for the clarifying comment regarding citing previously published works recommended by the reviewers.

We have carefully reviewed and evaluated each suggested publication. We found the references provided by the reviewers to be highly valuable and relevant to our study. As a result, we have incorporated several of them into the revised manuscript, as detailed in our point-by-point responses to the individual reviewer’s comments.

This inclusion has helped improve the context and strengthen the discussion of our work. We confirm that we have not cited any works solely based on the recommendation, but only those that we independently judged to be directly pertinent to our research.

All relevant citations have been added to the manuscript and are included in the updated reference list.

Additional Editor Comments:

Dear Authors,

Thank you for submitting your manuscript entitled “Diet and density effects on growth, proximate composition and fatty-acid profile of Hediste diversicolor juveniles” to the journal. The manuscript has now been evaluated by external peer reviewers and assessed at the editorial level.
The reviewers have raised several substantial concerns that must be carefully and comprehensively addressed before the manuscript can be reconsidered. Detailed reviewer comments are provided below to guide you in revising the manuscript.
upon submitting a revised version, please ensure that all reviewer comments are addressed point-by-point in a separate response document.
Thank you for considering the journal for your work. We look forward to receiving your revised submission.

[Note: HTML markup is below. Please do not edit.]

Answer: Dear Dr. Zahran and the PLOS ONE Editorial Team,

Thank you for the opportunity to revise and resubmit our manuscript, **“Diet and density effects on growth, proximate composition and fatty-acid profile of *Hediste diversicolor* juveniles.”**

We sincerely appreciate the time and effort invested by you and the external reviewers in evaluating our work. We have carefully studied all the comments and critiques provided.

We are submitting a thoroughly revised version of the manuscript, in which we have addressed every point raised by the reviewers comprehensively and point-by-point. The revisions have included additional data analysis, clarifications of methodology, expanded discussions, and textual edits to improve clarity and scientific rigor. We believe these changes have significantly strengthened the manuscript.

As requested, we have prepared the following documents for resubmission:

1. A **point-by-point response** to all reviewer comments (uploaded as a separate “Response to Reviewers” file).
2. A **marked-up copy** of the revised manuscript with all changes highlighted.
3. A **clean version** of the final manuscript.

We are grateful for the constructive feedback and hope that our revised manuscript now meets the high publication standards of PLOS ONE. Thank you again for your consideration.

Sincerely,

Reviewer's Responses to Questions

**Comments to the Author**

1. Is the manuscript technically sound, and do the data support the conclusions?

The manuscript must describe a technically sound piece of scientific research with data that supports the conclusions. Experiments must have been conducted rigorously, with appropriate controls, replication, and sample sizes. The conclusions must be drawn appropriately based on the data presented.

Reviewer #1: Yes

Reviewer #2: Yes

2. Has the statistical analysis been performed appropriately and rigorously?

Reviewer #1: Yes

Reviewer #2: No

3. Have the authors made all data underlying the findings in their manuscript fully available?

The [PLOS Data policy](http://www.plosone.org/static/policies.action#sharing) requires authors to make all data underlying the findings described in their manuscript fully available without restriction, with rare exception (please refer to the Data Availability Statement in the manuscript PDF file). The data should be provided as part of the manuscript or its supporting information, or deposited to a public repository. For example, in addition to summary statistics, the data points behind means, medians and variance measures should be available. If there are restrictions on publicly sharing data—e.g. participant privacy or use of data from a third party—those must be specified.

Reviewer #1: Yes

Reviewer #2: Yes

4. Is the manuscript presented in an intelligible fashion and written in standard English?

PLOS ONE does not copyedit accepted manuscripts, so the language in submitted articles must be clear, correct, and unambiguous. Any typographical or grammatical errors should be corrected at revision, so please note any specific errors here.

Reviewer #1: Yes

Reviewer #2: Yes

5. Review Comments to the Author

Please use the space provided to explain your answers to the questions above. You may also include additional comments for the author, including concerns about dual publication, research ethics, or publication ethics. (Please upload your review as an attachment if it exceeds 20,000 characters)

**Reviewer #1:** Introduction
Line 84 to 93 – These two sentence of the this paragraph is unnecessary. Remove it. Instead of this, importance of live prey utilization in both marine and freshwater fish species should have been summarized with using related references (see below). The authors should go for alternative live preys such as polycheates utilization and its importance. Then you can continue fatty acid composition of this polyceates.
Eryalcin, K. (2018). Effects of different commercial feeds and enrichments on biochemical composition and fatty acid profile of rotifer (Brachionus Plicatilis, Muller 1786) and Artemia franciscana. Turkish Journal of Fisheries and Aquatic Sciences, 18.
Turcihan, G., Turgay, E., Yardımcı, R. E., & Eryalçın, K. M. (2021). The effect of feeding with different microalgae on survival, growth, and fatty acid composition of Artemia franciscana metanauplii and on predominant bacterial species of the rearing water. Aquaculture International, 29(5), 2223-2241.
Eryalçın, K. M. (2019). Nutritional value and production performance of the rotifer Brachionus plicatilis Müller, 1786 cultured with different feeds at commercial scale. Aquaculture International, 27(3), 875-890.
Turcihan, G., Isinibilir, M., Zeybek, Y. G., & Eryalçın, K. M. (2022). Effect of different feeds on reproduction performance, nutritional components and fatty acid composition of cladocer water flea (Daphnia magna). Aquaculture Research, 53(6), 2420-2430.

Answer: We thank the reviewer for this constructive suggestion. In response, we have removed the two sentences indicated (original manuscript lines 84–93) and replaced them with a concise, evidence-based summary of (1) the nutritional importance of live prey for marine and freshwater larvae, (2) the role of feed/enrichment in determining rotifer and *Artemia* biochemical and HUFA profiles, and (3) the potential of polychaetes (*Hediste diversicolor*) as an alternative live prey and vehicle for HUFA delivery. We added citations to support these points (Eryalçın 2018; Turcihan et al. 2021; Eryalçın 2019; Turcihan et al. 2022) and recent studies describing the fatty-acid composition and enrichment dynamics of *H. diversicolor* (Wang et al., 2019). We then continued the manuscript with a focused section on polychaete fatty-acid composition, as requested. The exact replacement text and the newly added references are provided in the revised manuscript.

Line 97 – The fatty acid metabolism of H. diversicolor should have been explored. What are the differences comparing rotifer, artemia ? Please see related refences than you can get some clues (see below).
Villena-Rodríguez, A., Monroig, Ó., Hontoria, F., Malzahn, A. M., Hagemann, A., & Navarro, J. C. (2025). Effects of temperature and salinity on the LC-PUFA biosynthesis and composition of the nereid polychaete Hediste diversicolor fed side streams. Aquaculture International, 33(1), 56.
Villena-Rodríguez, A., Navarro, J. C., Varó, I., Aguado-Giménez, F., Pérez-Ara, J., Lizanda, M., ... & Monroig, Ó. (2025). Investigating the potential of dietary iron supplementation to enhance long-chain polyunsaturated fatty acid biosynthesis in Hediste diversicolor. Scientific Reports, 15(1), 42473.

Answer: We thank the reviewer for this suggestion. We have removed the originally redundant sentence and replaced it with a concise paragraph summarizing current knowledge on LC-PUFA biosynthesis in *Hediste diversicolor*, contrasting it with rotifers and *Artemia*, and noting the modulatory roles of temperature, salinity and micronutrients (Villena-Rodríguez et al., 2025 a,b). We also added a bridging sentence clarifying that we evaluate how these metabolic capacities affect proximate and fatty-acid composition under the culture conditions used in this study.

Line 100 – There should be mentioned specifically PUFAs importance in different stage of fish. That means larvae, growth out and broodstock. Each life stage of fish requiere different amount of those fatty acids. The authors should define with related references. This is the important part of study. It should be extended.
Eryalçın, K. M., Ganuza, E., Atalah, E., & Hernández Cruz, M. C. (2015). Nannochloropsis gaditana and Crypthecodinium cohnii, two microalgae as alternative sources of essential fatty acids in early weaning for gilthead seabream. Hidrobiológica, 25(2), 193-202.
Tseng, Y., Eryalçın, K. M., Sivagurunathan, U., Domínguez, D., Hernandez-Cruz, C. M., Boglione, C., ... & Izquierdo, M. (2023). Effects of the dietary supplementation of copper on growth, oxidative stress, fatty acid profile and skeletal development in gilthead seabream (Sparus aurata) larvae. Aquaculture, 568, 739319.

Answer: We thank the reviewer for this important suggestion. We have extended the Introduction to explicitly discuss the functional importance of LC-PUFA (EPA, DHA and ARA) at different ontogenetic stages (larvae, grow-out/juveniles and broodstock), and we now cite key references that quantify these stage-specific requirements and their physiological roles. Specifically, we note the high HUFA and phospholipid needs of early larvae, the EPA+DHA thresholds linked to sustained growth in juveniles and grow-out fish, and the influence of EPA/DHA/ARA balances on broodstock reproductive performance; we also cite Eryalçın et al. (2015) and Tseng et al. (2023) to connect microalgal and micronutrient effects to larval fatty-acid outcomes. The revised text clarifies how these stage-specific PUFA targets inform our evaluation of *Hediste diversicolor* as a live prey and the relevance of its modifiable FA profile. The revised manuscript lists the added citations. We appreciate the reviewer’s guidance and believe the added material strengthens the manuscript’s framing of fatty-acid relevance across life stages.

Material and Methods
Line 129 - I advice to put subcaption such as ’’Polyceate Husbandry’’?

Answer: We thank the reviewer for this helpful suggestion. We have added a new subsection entitled “Polychaete husbandry” to clearly distinguish the description of *Hediste diversicolor* culture conditions and management from the surrounding text. This improves the structure and readability of the manuscript.

Line 164 – The labeling groups should be changed. This can be like Commercial C1, C2, C3, C4 depending on density. And other can be like Waste (W1, W2, W3, W4). In that way, similarity can be removed among treatments. Specification of commercial diets should be given (brand, country, size).

Answer: We thank the reviewer for this helpful suggestion. The treatment labels have been revised to improve clarity and avoid similarity among experimental groups. Polychaetes fed a commercial diet are now designated as C1, C2, C3 and C4, while those fed sturgeon effluent are designated as W1, W2, W3 and W4, with the numerical index indicating increasing stocking density. In addition, the commercial diet has been fully specified in the revised manuscript, including brand name, manufacturer, country of origin and particle size.

Line 173 to 181 – This paragraph can be removed its because you are already explaining zootechnical measurements (even we don’t call it like that)

Answer: We thank the reviewer for this observation. The paragraph was redundant with descriptions provided elsewhere in the Methods section. Following the reviewer’s suggestion, this paragraph has been removed in the revised manuscript.

Line 202 - How it has been measured and with which kind of tools, brand, country and model should be given.

Answer: We thank the reviewer for this valuable suggestion. The manuscript has been revised to include detailed information on the measurement of water flow rate. Specifically, we now state that flow rate was monitored using an inline ultrasonic flowmeter, including the model, manufacturer, and country of origin, and that measurements were independently verified by weekly volumetric calibration. This information has been added to the Materials and Methods section to improve methodological transparency and reproducibility.

Results
I think two caption should be get together in one subcaption like ‘’Growth performance and Survival of H. diversicolor’’. Same comment for Proximate composition section. Proximate composition of H.diversicolor should be combined both ‘’…fed commercial feed’’ and ‘’….. fed sturgeon waste’’; combined under one caption.

Answer: We thank the reviewer for the suggestion. The captions for survival and growth have been combined into a single subcaption: “Growth Performance and Survival of *H. diversicolor*.” Similarly, the proximate composition data for commercial feed and sturgeon waste treatments have been merged under one caption: “Proximate Composition of *H. diversicolor* Across Feeding Treatments.”

Line 319 – Proximate and Fatty acid composition of sturgeon waste and commercial diets should be given first.

Answer: We thank the reviewer for this comment. In the revised manuscript, the proximate and fatty acid composition of the sturgeon waste and commercial diets has been presented first, prior to reporting the composition of *H. diversicolor*, to improve clarity and logical flow of the Results section.

Line 333 - While mentioning ‘’Fatty acid profiles of H.diversicolor’’ at between Line 326 and 336, ARA, EPA and DHA values should be given.

Answer: We thank the reviewer for this suggestion. The Results section has been revised to include the explicit values for ARA (C20:4n-6), EPA (C20:5n-3), and DHA (C22:6n-3) for all stocking densities, including standard deviations, and comparisons among treatments. This provides a complete and transparent view of the long-chain PUFA composition of *H. diversicolor* (see revised text and Table 6).

Line 335 – This paragraph should go up.

Answer: We thank the reviewer for the suggestion. The concluding sentence regarding the optimal intermediate stocking density (T1-2) has been moved to the beginning of the paragraph, so the main finding is presented before the detailed fatty acid data.

Discussion
I don’t know whether PlosOne includes subcaptions or not at Discussion section. Please check !

Answer: Thanks, revised.
I think, both tables (Table 1 and Table 2) can be combined.
Same comment for Table 3 and Table 4.

Answer: Thank you, we agree. Tables 1 and 2 (growth and survival) as well as Tables 3 and 4 (proximate composition) have been combined into single tables for each section (now Table 1 for growth and survival, Table 3 for proximate composition) to facilitate direct comparison between commercial feed and sturgeon waste treatments. Statistical significance letters have been retained, with lowercase letters for commercial feed groups and uppercase letters for waste feed groups.
At Table 5, you can use upper superscript letters just like you did in other tables.
Please correct ‘’Sat’’ to ‘’Saturated’’ both fatty acid tables.
At Table 8 – Use upper superscript letters instead of ‘’*’’

Answer: Thank you for the suggestions. Superscript letters have been added to Table 5 and Table 8 to indicate statistically significant differences, consistent with the other tables. Additionally, “Sat” has been corrected to “Saturated” in both fatty acid tables.

**Reviewer #2:** Both the presentation of results and the statistical analysis require improvement.

Answer: Thanks, we revised the result based on your valuable comment.

Figure 1 – In the figure, it is not clear that each experimental group corresponds to three tanks. This information should be clearly represented. Additionally, the terminology corresponding to the tested densities (i.e. 1, 2, 3 and 4) should be included.

Answer: Thank you for the comment. Figure 1 has been revised to clearly indicate that each experimental group consisted of three replicate tanks. In addition, density codes (D1–D4) corresponding to the tested stocking densities have been added to the figure and caption for clarity.

Line 179 – What was the feed-to-worm biomass ratio used in this study?

Answer: We thank the reviewer for pointing out this omission. The feed-to-worm biomass ratio has now been specified in the Methods section. Polychaetes were fed daily at 1% of their total wet biomass, divided into two meals administered at 08:00 and 15:00 h. This information has been added to the “Polychaete culture and experimental design” subsection.

Proximate composition analysis: How was ash content determined?

Answer: We thank the reviewer for the comment. Ash content was determined by incinerating the dried samples in a muffle furnace at 550 °C for 6 hours, and the remaining inorganic residue was weighed to calculate the ash percentage.

Lines 222 and 228 – Total lipids were determined using two different methods (Soxhlet and Folch). Please justify the use of two methodologies and clarify which method corresponds to the results presented.

Answer: We thank the reviewer for this comment. Total lipids were determined using the Soxhlet extraction method for proximate composition analysis, providing the quantitative lipid content reported in the manuscript. For fatty acid profiling, lipids were extracted using the Folch method, which is widely recognized for preserving the integrity of individual fatty acids during transesterification and GC analysis. Using these two complementary methods allows accurate determination of both total lipid content and detailed fatty acid composition from the same samples.

Line 238 – Please specify the FAME standards used.

Answer: We thank the reviewer for the comment. Individual FAMEs were identified by comparing their retention times with those of authenticated FAME standards, including C14:0 (myristic acid), C16:0 (palmitic acid), C16:1 (palmitoleic acid), C18:0 (stearic acid), C18:1 (oleic acid), C18:2 (linoleic acid), C18:3 (linolenic acid), and C20:4 (arachidonic acid), obtained from Sigma-Aldrich, China.

Line 240 – Please confirm whether fatty acid results are expressed as mg FA per g of wet tissue and explain the calculation. Tables 6, 7 and 8 present fatty acid data as a percentage of total fatty acids. How do you justify this discrepancy in units?

Answer: We thank the reviewer for this comment. Fatty acid concentrations in *H. diversicolor* were initially determined as mg fatty acid per g of wet tissue (mg/g) using peak integration and an internal standard. For presentation in Tables 6–8, the absolute values were converted to percentages of total fatty acids (% of total FA) using the formula: % FA = (individual FA / sum of all quantified FA) × 100, which allows clear comparison of fatty acid profiles across treatments and highlights compositional differences in SFA, MUFA, and PUFA. High-purity helium was used as the carrier gas, and individual FAMEs were identified by comparing their retention times with authenticated FAME standards, including C14:0, C16:0, C16:1, C18:0, C18:1, C18:2, C18:3, and C20:4 (Sigma-Aldrich, China). Absolute mg/g values provide information on tissue deposition, whereas percentages reflect fatty acid composition, and both sets of data are complementary for interpreting nutritional and biochemical results.

Line 268 – The identification of the experimental group with a density of 5000 ind./m² (T1–3) must be included.

Answer: We thank the reviewer for this comment. The text has been revised to clearly identify the experimental group, and T1–3 (5000 ind./m²) is now explicitly indicated as the optimal density for maximizing biomass yield.

Lines 298–310 and Tables 3 and 4 –
The sum of moisture, protein, lipids and ash is close to 100%. Does H. diversicolor not contain carbohydrates? Are these results consistent with previous studies?

Answer: We thank the reviewer for raising this important point. In our proximate composition analysis, we measured moisture, crude protein, crude fat, and ash; we did not directly measure carbohydrates. The sum of moisture, protein, lipids, and ash is therefore close to 100% because carbohydrates in *Hediste diversicolor* are typically present at very low concentrations and are not quantified directly in standard proximate analyses. Previous research on the biochemical composition of *H. diversicolor* and similar polychaetes has shown that carbohydrates often represent a negligible component of tissue composition and are frequently calculated by difference rather than measured directly. For example, proximate studies of *H. diversicolor* reared under different diets reported high protein and lipid fractions with carbohydrate content being minor or not separately reported, indicating that the dominant biochemical fractions in this species are moisture, protein, fat, and ash, consistent with our findings (Wang et al., 2019). <https://doi.org/10.1016/j.aquaculture.2018.12.047>

Additionally, the proximate composition of H. diversicolor obtained with the two different feeds is not compared. The information presented in Tables 3 and 4 should be compiled into a single table. For each tested density, results obtained with the two diets should be directly compared, including appropriate statistical analysis.

Answer: We thank the reviewer for this important suggestion. In the revised manuscript, we have consolidated the proximate composition data for *Hediste diversicolor* fed commercial (C) and sturgeon waste (W) diets into a single table. For each stocking density, the results obtained with the two diets are now directly compared, and significant differences between diets at the same density are indicated using independent-samples t-tests (P < 0.05). This approach ensures that both within-density diet effects and density effects within each diet are clearly presented, allowing a more direct comparison of the influence of dietary source on moisture, crude protein, crude fat, and ash content. The revised Table 3 now includes all densities and both diets, with asterisks indicating statistically significant differences between diets at the same density. Corresponding updates were made to the Results and Methods sections to reflect these comparisons. We believe this revision fully addresses the reviewer’s comment by providing direct, statistically supported comparisons between the two feeding regimes at each density.

Lines 311–324 / Table 5 and Figure 3 –
Information regarding the proximate composition of the two experimental feeds is duplicated. Table 5 and Figure 3 present the same data; therefore, Figure 3 should be removed.

Answer: We thank the reviewer for this observation. The proximate composition data of the two experimental feeds were indeed presented redundantly in Table 5 and Figure 3. To avoid duplication and improve clarity, Figure 3 has been removed, and the information is now presented only in Table.
Moreover, the sum of moisture, protein, fat and ash represents only 53.51% and 72.21% of beluga sturgeon by-products and beluga sturgeon feed, respectively. What does the remaining fraction correspond to? Carbohydrates, including fibre? Could differences in these nutrients also help explain differences observed in polychaete performance?

Answer: We thank the reviewer for this important observation. The proximate components reported in Table 5 (moisture, crude protein, crude fat, and ash) do not sum to 100% because carbohydrates/fibre were not measured directly. The remaining fraction (46.49% for beluga sturgeon by-products; 27.79% for beluga sturgeon feed) most likely corresponds to nitrogen-free extract (soluble carbohydrates such as starches and sugars), crude fibre (insoluble carbohydrates), and other non-protein organic material (binders, carbohydrates, minor additives). Differences in this fraction between the two feeds could indeed influence digestibility and nutrient utilisation, and therefore may contribute to the observed differences in polychaete performance. However, direct measurement of crude fibre/NFE was not performed in the present study. We have updated Table 5 and its legend to report the calculated “remaining fraction (100 − sum of reported proximate components)” and added a short note in the Results to clarify the likely composition and its potential implications.

Lines 338–367 –
The fatty acid profiles of H. diversicolor obtained with the two feeds should be compared for each density, including statistical analysis.

Answer: We thank the reviewer for this valuable comment. In response, we have revised the fatty acid analysis to include direct comparisons between the two diets at each stocking density. The fatty acid profiles of *Hediste diversicolor* fed commercial (T1) and sturgeon byproduct (T2) diets were compared within each density using independent-samples t-tests (P < 0.05). The results of these analyses are now presented in a revised combined table, where statistically significant differences between diets at the same density are clearly indicated by asterisks. In addition, a new subsection has been added to the Results section explicitly describing these diet-dependent differences in fatty acid composition across densities. The Methods section and table legend were also updated to clearly describe the statistical approach used for these comparisons. We believe these revisions fully address the reviewer’s concern by providing direct, statistically supported comparisons of fatty acid profiles between the two feeding regimes at each stocking density.

Lines 401–405 –
The data supporting these conclusions are not adequately highlighted in the Results section. Please refer to the comments regarding Tables 3 and 4.

Answer: We thank the reviewer for this valuable comment. In the revised manuscript, Tables 3 and 4 have been consolidated into a single table, presenting the proximate composition of *Hediste diversicolor* under both commercial (C) and sturgeon waste (W) feeding regimes across all stocking densities. For each density, direct comparisons between the two diets were performed using independent-samples t-tests (P < 0.05), and significant differences are now clearly indicated with asterisks in the table. The Results and Discussion sections have been updated accordingly to explicitly highlight these diet-dependent differences. We believe these revisions now provide a clear and statistically supported comparison between the two feeding regimes, addressing the reviewer’s concerns.

6. PLOS authors have the option to publish the peer review history of their article ([what does this mean?](https://journals.plos.org/plosone/s/editorial-and-peer-review-process#loc-peer-review-history)). If published, this will include your full peer review and any attached files.

If you choose “no”, your identity will remain anonymous but your review may still be made public.

**Do you want your identity to be public for this peer review?** For information about this choice, including consent withdrawal, please see our [Privacy Policy](https://www.plos.org/privacy-policy).

Reviewer #1: No

Reviewer #2: No

[NOTE: If reviewer comments were submitted as an attachment file, they will be attached to this email and accessible via the submission site. Please log into your account, locate the manuscript record, and check for the action link "View Attachments". If this link does not appear, there are no attachment files.]

To ensure your figures meet our technical requirements, please review our figure guidelines: https://journals.plos.org/plosone/s/figures

You may also use PLOS’s free figure tool, NAAS, to help you prepare publication quality figures: https://journals.plos.org/plosone/s/figures#loc-tools-for-figure-preparation.

NAAS will assess whether your figures meet our technical requirements by comparing each figure against our figure specifications.
